# “Not shifting, but sharing”: stakeholders' perspectives on mental health task-shifting in Indonesia

**DOI:** 10.1186/s12912-022-00945-8

**Published:** 2022-06-24

**Authors:** Ferry Efendi, Gading Ekapuja Aurizki, Ah Yusuf, Lisa McKenna

**Affiliations:** 1 Faculty of Nursing, Universitas Airlangga, Campus C Mulyorejo, Surabaya, East Java 60115 Indonesia; 2 Community, Family and Gerontological Nursing Research Group, Universitas Airlangga, Surabaya, Indonesia; 3 Mental Health Nursing Research Group, Universitas Airlangga, Surabaya, Indonesia; 4 School of Nursing and Midwifery, La Trobe University, Melbourne, Victoria Australia

**Keywords:** Health worker, Mental health, Nurses, Primary care, Psychiatrists

## Abstract

**Background:**

Task-shifting, the distribution of tasks among health workers to address health workforce shortage, has been widely used to tackle mental health treatment gaps. However, its implementation in Indonesia has still been rarely explored. This study aimed to explore stakeholders’ perspectives on the implementation of mental health task-shifting to nurses in Indonesia's primary health care.

**Methods:**

An exploratory descriptive approach using in-depth interviews and focused group discussions (FGDs) was used. The study involved 19 stakeholders from the government's ministry directorates, professional organisations, and mental health practitioners. Thematic analysis was used to analyse the data.

**Results:**

Three themes emerged namely, task-shifting feasibility and acceptability, shared task implementation, and nurse role enhancement issues, with 14 sub-themes.

**Conclusions:**

Task-shifting on mental health issues in the eye of Indonesian stakeholders is viewed as a matter of sharing and collaboration. Implementation of task-shifting in Indonesia may require policies in place and political will across stakeholders. Further scrutiny on task-shifting implementation is needed by considering the local context and national environment.

## Background

Mental disorders are one of the main factors contributing to global burden of disease [1]. People with mental disorders have increased risk of all cause mortality, as well as substantial reduction in life expectancy between 10 and 20 years [2]. Moreover, the COVID-19 pandemic has exacerbated mental health problems among the general population [3], yet many mental health problems are not adequately addressed [4]. This situation leads to a situation referred to as the 'treatment gap', which is the difference between the number of people suffering from a disease and those receiving treatments [5, 6].

The treatment gap shows disparity in mental health services between developed and developing countries [7], mainly related to scarcity, limited access, and inefficiency in the use of resources in mental health services [8, 9]. The WHO World Mental Health Survey (WHO-WHMS) revealed that more than half of people with mood, anxiety, and substance abuse disorders from 25 countries did not get the treatments they needed [10]. Overall, the gap in high-income countries reached 63.2% and even wider gaps were found in the upper- and lower-middle-income categories with 78% and 86.3%, respectively [10].

Numbers of people with mental disorders in Indonesia are predicted to rise from around 2.58 million in 2017 to around 2.99 and 3.24 million in 2020 and 2024 respectively [11]. During the pandemic, rates of anxiety, depression, and trauma among the Indonesian population were reportedly around 70% across 34 provinces [12]. The provision of mental health services in Indonesia's community health centres (widely known as *puskesmas*) can be dated back to the late-1960s, through deployment of psychiatrists [13], which was then shifted to trained general practitioners and nurses since the 1990s [14] and for some regions, clinical psychologists since the mid-2000s [15]. However, the country only has 0.31 psychiatrists, 0.17 clinical psychologists, and 2.52 trained mental health nurses per 100,000 people [16]. These numbers are considered insufficient to serve the population of around 270 million people, especially when the majority of those specialists are still concentrated in Java Island [15].

One strategy that has emerged to address treatment gaps is task-shifting, involving non-specialist health workers providing mental health services [17–19]. Since producing specialists takes time and is a long-term investment, training available health workers can improve a health system in the short to medium term and save costs [20]. Nurses are often involved in task-shifting because they constitute the largest group of health professionals, have good capabilities and are available in almost every health care facility [21].

In Indonesia, task-shifting to nurses had been tested around two decades ago but its implementation was not as successful as expected. In early 2000, the Indonesian Medical Association (IMA) and Indonesian National Nurses Association (INNA) agreed to implement a task-shifting program to enhance health care service provision, however, the agreement between the two professional organisations was cancelled due to interprofessional conflicts in some regions [22]. Recently, task-shifting has been considered in addressing some health issues in the country, including child health [23], HIV/AIDS [24], mental health [25, 26], and COVID-19 [27]. The IMA considered task-shifting as a temporary solution while waiting for the required number of medical personnel to be fulfilled [28]. In addition, since 2014, task-shifting has been regulated with Health Worker and Nursing Laws [29, 30], which permit nurses or other health workers to perform limited medical actions in the absence or shortage of medical personnel.

Research on mental health task-shifting has been conducted in developed and developing countries with promising results [31–41]. Nevertheless, studies focused on mental health task-shifting in Indonesia are very limited. Among others, a randomised controlled trial conducted in some *puskesmas* in Yogyakarta concluded task-shifting involving trained general practitioners and nurses could effectively manage mild to moderate mental health cases [25]. However, a cross-sectional study found task-shifting was not effective because, compared to psychiatrists and psychologists, non-specialist practitioners did not have capacities to withstand stigmatised views of patients with mental health problems and implement evidence-based mental health practices [26]. These findings reflect the existing opposing views on task-shifting implementation in Indonesia, which to date have barely been explored. Therefore, this study aimed to explore stakeholders’ perspectives on the implementation of mental health task-shifting to non-specialist health workers, especially nurses, in Indonesia's primary health care setting.

## Methods

This qualitative study used an exploratory descriptive approach [42], using in-depth interviews and focused group discussions (FGDs). A question that guided this study was: “What do stakeholders think about shifting mental health interventions normally provided by mental health specialists to non-specialist providers, especially nurses, in primary health care settings?”

Regarding the specific tasks included in task-shifting, this study referred to the World Health Organisation (WHO) mental health Gap Action Programme (mhGAP) – version 2.0 [43]. The list of tasks included in mhGAP was quite broad, including procedures that can only be undertaken by medical personnel in normal circumstances such as diagnosis and pharmacological interventions. However, as an exploratory study, this study did not specify these procedures from the beginning and was more focused on the participants’ views regarding what they perceived about task-shifting phenomenon in Indonesia.

This study used data source triangulation to ensure broad perspectives and voices were obtained by involving participants from different organisations and professions [44, 45]. The investigators invited key stakeholders to take part, including the Ministry of Health's directorates, professional organisations and mental health practitioners. The choice of the invitees was made by consensus among investigators considering their proximity to task-shifting and mental health issues, institutional representations, as well as availability during data collection.

The interviews and FGDs were led by two team members with nursing backgrounds and conducted in Bahasa Indonesia. As the investigators were all nurses, unconscious bias could occur in the form of directed questions and interpretations that may favour the involvement of nurses in the provision of mental health care.

Data collection was conducted from May to November 2021 in Jakarta (3 FGDs and 1 individual interview) and online Zoom meeting (7 interviews), due to the increasing COVID-19 cases in Indonesia Table 1. The data collection activities were postponed from July to September following the implementation of Community Activities Restrictions Enforcement.

**Table 1:** List of participants for task-shifting research

Participant	Gender	Institution	Level	Data Collection
P1	Female	Center for Planning and Management of Human Resources for Health, BPPSDMK, Ministry of Health (MoH-CPMHRH)	National	FGD (in person)
P2	Female			
P3	Female			
P4	Female			
P5	Female			
P6	Female	Directorate of Mental Health and Drugs Prevention and Control, Ministry of Health (MoH-Mental Health)	National	Individual interview (in person)
P7	Female	Directorate of Primary Health Service, Ministry of Health (MoH-Primary Health Service)	National	FGD (in person)
P8	Male			
P9	Female			
P10	Female	Indonesian National Nurses Association (INNA)	National	FGD (in person)
P11	Male			
P12	Male			
P13	Female	Indonesian Mental Health Nurses Association (IPKJI)	National	Individual interview (online)
P14	Male		Provincial	
P15	Female	Mental health nurses at community health centres (*puskesmas*) in Bali Province	Clinical	
P16	Female			
P17	Female			
P18	Female			
P19	Female			

The investigators developed semi-structured questions focused on five topics: 1) stakeholders' perspectives on mental health treatment gaps and the situation of mental health services in Indonesia's primary health care; 2) strategies implemented to reduce disparities in mental health services; 3) stakeholders' perspectives on task-shifting and its implementation; 4) prerequisites that must be met to implement task-shifting; and 5) other issues related to mental health services in Indonesia. The contents of interviews and FGDs were audio-recorded, transcribed verbatim, and translated by the team members into English before the analysis commenced.

Thematic analysis was used to analyse the data. This analysis is appropriate to identify, analyse, and find patterns on qualitative data that require low interpretation levels [46]. The investigators imported the data to NVivo to identify codes through iterative reading and re-reading of the transcripts. Codes were grouped to sub-themes and themes based on the similarity of topics addressed. The team held a series of discussions to generate themes until a consensus was reached. The findings were checked by the participants' representatives to ensure trustworthiness.

## Results

Nineteen key stakeholders were interviewed representing three Ministry of Health organisations and directorates, two professional nursing organisations, and five community mental health nursing practitioners Table 1. Representatives from IMA and Indonesian Psychiatric Association (PDSKJI) were invited by email but did not respond.

Three themes emerged, namely task-shifting feasibility and acceptability, shared task implementation, and nurse role enhancement issues, with 14 sub-themes Table 2.

**Table 2:** Summary of themes and sub-themes generated

Themes	Sub-themes
Theme 1: Task-Shifting Feasibility and Acceptability	Legal framework
	Contextual dependability
	In-service training
	Various views on task-shifting
Theme 2: Shared Task Implementation	Collaborative and coordinated care
	Staged and referral services
	Communications technology innovation
	Intertwined and complementary roles
Theme 3: Nurse Role Enhancement Issues	Overwhelming administrative and other tasks
	Lack of delegation standards
	Interprofessional bargain
	Nurse competence boundaries
	Unequal training distribution
	Inadequate national-level supports

### Task-shifting feasibility and acceptability

Before being implemented, participants identified task-shifting required some aspects to be met, such as the legal framework, appropriate contexts, in-service training provision, and acceptability from related stakeholders.

#### Legal framework

Some participants mentioned Nursing Laws (No. 38th of 2014) as the legal basis for the implementation of task-shifting to nurses.

*"Regarding task-shifting, the main regulation is Nursing Laws in article 35, which regulates how to deal with emergencies, how nurses must provide services. This is detailed in the 26th Minister of Health Regulation [of 2019] on the implementation of these Nursing Laws. It is written that facilities that do not have doctors or medical personnel, [nurses] are granted the authority, or so-called 'mandate'."* (P7, MoH-Primary Health Service)

*"There are some being delegated to nurses and indeed it has been regulated by laws. In the Nursing Laws, there's so called authority delegation."* (P12, INNA)

#### Contextual dependability

Task-shifting implementation was seen to be limited by contexts, such as issues, place, and time. If the context is no longer relevant, the authority granted to nurses can be revoked.

*"The [task-shifting] form would be an assignment from the head of regional health office, which would be adjusted to the needs of each region. Even the time is also adjusted. For example, if later there is a doctor, the decree will be revoked. If it is no longer needed, there is no more delegation."* (P7, MoH-Primary Health Service)

*"Task-shifting is a delegation caused by a big issue, such as high maternal and infant mortality rate. So it must be triggered by a big issue. For other issues, not yet. Well, for areas like villages where people are far away from doctors, simple [medical] treatment can be delivered [by nurses]."* (P5, MoH-CPMHRH)

#### In-service training

In-service training was identified as a prerequisite for task-shifting. Despite the shortage of medical personnel, task-shifting could only be conducted if nurses were capable to perform certain medical actions.

*"Ideally, they should have been given special training. If there are special needs [for mental health], ideally, it would be good if there is special briefing and training with a standardised curriculum."* (P8, MoH-Primary Health Service)

*"Counselling, according to the theory, is a specialist competence. But in Indonesia, it's permitted if the [non-specialist] counsellor has been trained. Abroad, only senior or specialist nurses can provide it. [If it needs to be delegated], in principal, they should be trained first."* (P11, INNA)

#### Various views on task-shifting

In Indonesia, task-shifting has yet to become mainstream strategy to tackle treatment gap because the policymakers did not see it as a promising option. Furthermore, task-shifting was still considered new and stakeholders held different opinions about its definition and implementation.

*"The concept [of task-shifting] is not yet determined. We're still looking for the [policy/program] format, so it's still uncertain. It is because the leaders' policy for task-shifting isn't there yet."* (P1, MoH-CPMHRH)

*"In Indonesia, the model is not task-shifting but collaboration among mental health professions in accordance with their respective duties and authorities. As for task-shifting, according to my understanding, the task that should be done by a psychiatrist is delegated to a general practitioner, then to a nurse, right? This is delegation. For me, building a system for mental health is not using task-shifting."* (P6, MoH-Mental Health)

*"The context that will be explored is whether task shifting is more specialised or basic for a nurse to recognise social psychiatry. We really hope, for remote areas or in provinces that do not have mental hospitals, nurses or their teams with doctors have been equipped with how to do initial therapy for patients suspected or having symptoms. [...] Don't let this not be handled just because there has never been any training, no mental hospitals, or no psychiatrists in that district."* (P7, MoH-Primary Health Service)

### Shared Task Implementation

Although the concept of task-shifting was relatively new for some stakeholders, health personnel arrangements in primary health care have long existed in practice. However, in mental health contexts, they were implemented more of a "shared" manner than the "shifted" ones.

#### Collaborative and coordinated care

Collaboration and coordination were described as often being used to strategise mental health specialist shortages, for which the tasks were shared among stakeholders considering their roles and competences, instead of being shifted to only one or two parties.

*"In reality, there is a lot of work that has been done by our colleagues in puskesmas, especially related to mental health and human resources. So, there are a lot of collaborative activities."* (P10, INNA)

*"I think civic engagement and task-shifting are interrelated concepts. Despite positive or good ideas, shifting the work and duties of health workers did not always run smoothly. Therefore, we should recognise the service users, from people with mental health disorders, their families or caregivers. Then we also know there are communities around them that are also stakeholders. Then, there are health workers, government, whether it's local at the village level, community level, or neighbourhood level."* (P13, IPKJI)

#### Staged and referral services

Some stakeholders believed that mental health services should be carried out in stages, not only between interdisciplinary teams but also across service levels.

*"The services should be levelled up to the end, to primary services, but still based on their authority in primary care. For instance, because the chronic condition is stable, by using a back referral system from mental health and psychosocial support, the patients can be treated there. If then they experience an exacerbation [...], the patients are referred [to the higher service level]."* (P6, MoH-Mental Health)

*"For mild common mental health disorders, the programs during home visits are educating families about mental health and how to find solutions if their complaints continue. If it's not successful, we will suggest to the family or patient themselves to visit the puskesmas. If in around one week the complaints still persist, then a referral will be given."* (P17, Primary Care Nurse)

#### Communications technology innovation

Communication technology was identified as enabling the implementation of task-shifting and task-sharing, so the specialist did not have to present in person to manage mental health cases and nurses could obtain direct and real-time supervision to perform the delegated tasks.

*"When they feel stuck or unable to do [the intervention], usually they will try to refer or ask other health workers from a remote location where the doctor is available. This is the reason for telemedicine, which allowed them to consult with other health workers who might understand more."* (P7, MoH-Primary Health Service)

*"There were some times when the patient experienced a condition that I couldn't solve on my own. So I asked the doctor to come immediately. Or sometimes via video call, I consulted with the doctor, either general practitioner or psychiatrist."* (P15, Primary Care Nurse)

#### Intertwined and complementary roles

Some stakeholders believed that mental disorders were complex problems and thus required interdisciplinary approaches. Nurses were seen to have unique roles and competences, as did doctors and other health professionals.

*"In primary care, the treatment was carried out by the team of doctors and nurses. The initial interview is conducted by a nurse, then the diagnosis is by a doctor. [After the doctor] gave therapy, medication and education, they are returned to the nurse again to be given nursing care. In my opinion, that is the ideal one. There are competencies that nurses do not have but general practitioners do. But there are also competencies that general practitioners do not have but nurses do."* (P6, MoH-Mental Health)

*"I could say medicine is indeed the [doctor's] domain, but it doesn't mean if we don't have medicine, we don't provide services. We still provide [nursing] care. [...] I always say that drugs can't make a person able to control hallucinations or to communicate or to socialise if we don't train them with care. I always emphasise that. Medicine is very important, but if there is no medicine, don't make the service unavailable."* (P14, IPKJI)

### Nurse Role Enhancement Issues

Nurses are identified as available from the top-level to community-based services. Therefore, nurses’ role enhancement was considered pivotal to strengthen the health system, especially in light of shortages of medical personnel. However, such enhancement was found to pose numerous challenges.

#### Overwhelming administrative and other tasks

Primary care nurses were often seen to be assigned to many programs and overwhelmed with administrative tasks.

*"The mental health program holder is only one person in every puskesmas, but they manage not only one program. So many programs need to be managed which makes them not focus on that one [mental health] program."* (P14, IPKJI)

*"We have to carry out several programs. I myself can handle three, apart from the main services at puskesmas. All of my colleagues are also like that. One person can handle two or three."* (P18, Primary Care Nurse)

#### Lack of delegation standards

Participants identified that nurses had raised concerned about the lack of delegation standards when they had to carry out medical interventions. To avoid legal problems, a written and documented delegation procedure is required to ensure the nurses' safety while performing actions beyond their usual level of authority.

*"There is a delegation of authority. So far, it hasn't been written yet, only a letter of assignment given to us which was brought by the psychiatrist. They immediately gave us orders to treat the mental health clients, so we just have to be ready. As there was already permission from the psychiatrist, [we felt] that also gave us the authority to act."* (P15, Primary Care Nurse)

*"I think the key word is: it should be in black and white, clearly from the top. And that's what we need. There is legalisation that nursing is indeed allowed to have the authority to do certain things."* (P13, IPKJI)

#### Interprofessional bargain

It was raised that task-shifting could not be carried out without involving and obtaining approval from the medical profession. However, to some extent, it was still seen to be difficult to get such approval as there was still a sense of competition among mental health professionals.

*"We can't say that we encourage it, because to issue a task-shifting policy is not easy. Because there are bargainings between professions, and determining boundaries on how far the competencies can be delegated is also complicated."* (P5, MoH-CPMHRH)

*"There’s a kind of sense of competition among mental health professionals, which ideally should be sit together.”* (P13, IPKJI)

#### Nurse competence boundaries

Participants recounted that recognition was something that nurses still fought for. Some stakeholders questioned whether nurses were capable of delivering the shifted tasks and what kind of interventions nurses could offer to treat people with mental health issues.

*“It’s different when the task shifting was related to, for example, nurses carrying out environmental health or nutrition services. Maybe the friction or problems that will arise [are minimum]. If it’s about medical treatment and so on, there are risks given the limited knowledge [of nurses] about medications or diagnosing a disease, causing them to be at risk of giving the wrong prescription.”* (P8, MoH-Primary Health Service)

*“In more developed countries, we know there are shared competences that can be done together. For example, a specialist therapy, cognitive behaviour therapy [CBT]. How about Indonesia? Therapies such as CBT should be carried out and recognised by all professional health workers, mental health workers, and also the community, as a competence that is mutually shared, including for nurses. I think that’s our task [to achieve that].”* (P13, IPKJI)

#### Unequal training distribution

Despite being pivotal, standardised mental health training was seen to not be widely available for nurses, especially in remote areas.

*“[In our some districts], all [mental health] nurses have been trained about CMHN [community mental health nursing], but in other districts there are none. So, it affects their ability to provide care."* (P14, IPKJI)

*“There is CMHN in some provinces, including East Java, but in other provinces it is not evenly distributed depending on the provincial government. So yes, we are already there [providing training], but how can it be leveled up until evenly distributed [to all regions]?”* (P13, IPKJI)

#### Inadequate national-level supports

The implementation of task-shifting and task-sharing was seen to require a myriad of supports both from government and professional organisations. However, the current supports were deemed to be insufficient to drive the implementation of task-shifting.

*“If you want the ideal, [the support] should be from the top, from the national level will be very strong. And in the process it certainly involves multi-professions. If the top said A, the bottom will be A, right? However, if from the top is not clear, it would not be A but given the authority to each provincial or regional government. They will make their own policies.”* (P13, IPKJI)

*“The ones who know best about regulations and the impact of unregulated interventions are professional organisations. It’s mandatory for professional organisations to advocate and lobby primary care units to encourage official and regulated delegation.”* (P14, IPKJI)

## Discussion

This study explored the perspectives of stakeholders on the implementation of mental health task-shifting in Indonesia with three key themes emerging: task-shifting feasibility and acceptability, shared task implementation, and nurses’ role enhancement issues.

### Theme 1: Task-shifting feasibility and acceptability

It was recognised that a number of aspects should be considered when implementing task-shifting. The first of these is a legal framework. The WHO has emphasised that task-shifting should be supported by appropriate health legislation and administrative regulation that enables checks and balances and ensures the safety of both patients and health workers involved [47]. Otherwise, task-shifting implementation can induce jurisdictional debates on nurses’ scope of practice [48]. In Indonesia, at least two laws have covered task-shifting topics: Health Workers and Nursing Laws. These laws grant permission to nurses and health workers to provide medical services in certain limited contexts in the absence of medical personnel. The implementation should consider the providers’ competence and authorisation from the regional government [29, 30]. Indonesia’s laws have generally regulated the task-shifting standards, including the requirements that must be fulfilled upon implementation. Technical guidelines, however, are still needed to make sure the task-shifting is implemented smoothly and sustainably.

The requirements set by the laws are also in line with the other aspects covered in this theme, namely appropriate contexts in which task-shifting is urgently needed, in-service training to enhance the providers’ competence, and acceptability from stakeholders. These aspects correspond with task-shifting implementation criteria recommended by a systematic review and an international Delphi study involving participants from the United States, South Africa, United Kingdom, Nigeria, India, and Australia, among others, trained health providers, existing health human resources shortage or inaccessibility, important health issues, and socially acceptable interventions [33, 49]. These requirements are needed to maintain quality and ensure effective and efficient implementation.

Regarding acceptability, stakeholders in this study had different attitudes on task-shifting. One stakeholder opposed task-shifting, given that they believed mental health services should be conducted collaboratively in accordance with each profession’s competence. Meanwhile, others supported the implementation citing that task-shifting is needed to make sure no one is left behind. This finding corresponds with some evidence from some countries in Africa and South Asia finding that stakeholders generally have various attitudes on task-shifting, either positive, negative, neutral, or even skeptical [33, 50]. Opposing views can be barriers to task-shifting implementation, particularly if they come from policymakers.

### Theme 2: Shared Task Implementation

Despite different views on task-shifting, participants in this study had similar perceptions about the collaborative nature of mental health services. This supports the use of so-called ‘task-sharing’, a term that is closely linked with task-shifting. Although both terms similarly involve redistribution of duties among health workers, task-shifting gives more emphasis on task delegation or transfer, while task-sharing focuses on the involvement of providers with different qualifications to complete the tasks [49]. An Indonesian-based grounded theory coined the term ‘connecting care’ to describe collaborative mental health service models that involve multiple stakeholders [51]. Therefore, we consider that task-sharing is generally more acceptable for most stakeholders in Indonesia compared to task-shifting.

Task-sharing is implemented based on the intertwined and complementary roles of mental health workers through some approaches, e.g., collaborative and coordinated care; staged and referral services; and communication technology utilisation. These approaches are supported by a literature review as evidence-based components that facilitate task-sharing [52]. Usually implemented within a system involving various care components, from specialist services to self-care, these approaches also correspond with the WHO pyramid framework designed to provide optimal mental health services [53, 54]. Furthermore, communication technology, such as phone calls or Whatsapp, plays a pivotal role in mediating collaboration and care delivery. A systematic review identified technology as a strategy to leverage the scope of mental health services [31]. Another study focusing on developing medical devices for task-shifting for health professionals in Ethiopia, Ghana, and Uganda revealed devices should be easy to use, safe, and effective, especially for target users, i.e., less specialised health workers [55]. Technology utilisation can improve agility and responsiveness of mental health services and allows task-shifting to be demanded.

### Themes 3: Nurses’ role enhancement issues

Task-shifting (and task-sharing) require nurses’ roles to be enhanced. However, this study found that the enhancement process faced numerous barriers. First, nurses had administrative and other task responsibilities, such as finance, medical record maintenance, nutrition, health promotion, and environmental health. Primary care nurses may not be able to provide optimum services if they are burdened with too many administrative tasks [56]. Second, nurse delegation procedures were unstandardised. Delegations that do not follow any protocol or standard can raise accountability problems and be detrimental to nurses [57]. In Indonesia’s context, nurses carrying out medical actions without written delegation can be considered a criminal case [58]. Therefore, nurses’ role enhancement should be supported by policies to reduce unnecessary burdens and develop standardised delegation protocols.

Third, participants raised serious concerns about nurses’ abilities in undertaking medical tasks and which tasks could be performed independently by nurses. These concerns were associated with the duration of training and scope of practice, particularly on diagnostics and therapeutics, which are considered insufficient to take on medical roles [59, 60]. Fourth, there is potential resistance from the medical profession regarding task-shifting implementation. Doctors were concerned that nurses would take their authority and threaten their jobs [61]. Therefore, doctors preferred nurses to carry out only non-medical tasks [60].

Fifth, nurses’ role enhancements were found to be hindered by unequal training distribution. In-service training determines task-shifting feasibility [31, 62, 63] and can improve the knowledge, skills, and confidence of non-specialist health workers to deliver mental health interventions [64]. However, training and supervision for mental health task-shifting were generally seen to be lacking in terms of duration and frequency [34]. Policymakers should provide regular training and supervision for nurses to improve their abilities in delivering mental health services.

Sixth, supports from national-level stakeholders was perceived by participants to be lacking. In general, the participation of nurses in the policy-making process is also still very limited [65]. Therefore, nurses need to be encouraged to be more involved in the policy-making process, both at clinical, local, and national levels. Compared to the medical profession, nurses were particularly seen to be lacking representation in policymaking institutions.

### Strength and limitations

This is the first qualitative study to explore task-shifting and task-sharing in Indonesia involving stakeholders from national to clinical levels and could be a reference for the development of the emerging approaches in Indonesia and other settings, especially low-and-middle income countries where mental health services are not widely available in primary care.

Besides those strengths, this study has several limitations. Multi-leveled participants involved made it difficult to find commonalities in their answers, especially among national-level participants. This situation was inevitable as these participants had different backgrounds, positions, and organisations with their respective roles and proximity to task-shifting and task-sharing issues. Data from primary care nurses was saturated after the third participant and interviews were stopped at the fifth. For other participants, the investigators did not wait until the data was saturated and stopped data collection after all invitees were interviewed, unless they were unavailable or not responding.

Although some stakeholders have medical backgrounds, official representatives from medical professional organisations could not be recruited in the given research period, particularly from the Indonesian Psychiatric Association, so this study could not capture their opinions. Besides, invitations were addressed to the organisation and position instead of the person. The organisation appointed their representatives, which made the investigators could not control the personal representations such as gender and professional background.

The investigators planned to involve clinicians from some regions to capture different perspectives. However, due to implementation of COVID-19 restrictions, only primary care nurses from Bali Province could be interviewed. Therefore, this study cannot capture clinical situations in broader contexts to enrich the data as mental health services in each region are likely to be different. The findings are limited to the Indonesian context that has specific circumstances regarding the availability of mental health services and the supporting systems. Implementation in other countries requires careful examination.

The three-month gap became an obstacle as it significantly changed the research plan set by the investigators. It also delayed the data collection, analysis and manuscript writing. As the funder had a strict reporting deadline to adhere, the investigators cannot sent the transcript and research findings to all participants to get appropriate member checking.

## Conclusions

Despite facing numerous challenges, task-shifting in mental health service delivery has been practised in Indonesia for many years, especially in collaborative and coordinated formats (i.e., task-sharing). Interprofessional collaboration across stakeholders is inevitable to ensure the best quality services of mental health care in community, particularly in rural and remote areas. This becomes more important when task-sharing has a more practical basis in Indonesia’s mental health services compared to task-shifting.

For future practice, the implementation of task-shifting or task-sharing of mental health interventions requires the involvement of highly skilled primary care nurses. Nurses have to improve their knowledge and skills in managing mental health patients through continuous training and education. Supporting laws and policies are pivotal for the sustainability of task-shifting and task-sharing. Nurses also need to consider legal aspects before accepting any delegated medical tasks, which are beyond their scope of practice, to avoid ethical or legal issues.

Further studies should assess the need to implement task-shifting or task-sharing from local leaders in very remote areas where mental health specialists are not available. The capacity of primary care nurses to undertake advanced mental health tasks also needs to be explored. This study can be initial guidance for nurses who have to undertake extended roles in mental health services in rural and remote settings.

## Data Availability

All relevant data are within the paper and its supporting information files.

## Abbreviations

CMHN: Community Mental Health Nursing
COVID-19: Coronavirus Disease 2019
IMA: Indonesian Medical Association
INNA: Indonesian National Nurses Association
IPKJI: Indonesian Mental Health Nurses Association
PDSKJI: Indonesian Psychiatric Association
Puskesmas: *Pusat Kesehatan Masyarakat* (Community Health Centre)
WHO-WHMS: World Health Organization World Mental Health Survey
